# Prediction of persistent shoulder pain in general practice: Comparing clinical consensus from a Delphi procedure with a statistical scoring system

**DOI:** 10.1186/1471-2296-12-63

**Published:** 2011-06-30

**Authors:** David Vergouw, Martijn W Heymans, Henrica CW de Vet, Daniëlle AWM van der Windt, Henriëtte E van der Horst

**Affiliations:** 1 Institute for Research in Extramural Medicine, VU University Medical Center Amsterdam, The Netherlands; 2 VU University, Institute for Health Sciences, Department of Methodology and Applied Biostatistics, Amsterdam, The Netherlands; 3 Primary Care Musculoskeletal Research Centre, Keele University, Keele Staffordshire, UK

## Abstract

**Background:**

In prognostic research, prediction rules are generally statistically derived. However the composition and performance of these statistical models may strongly depend on the characteristics of the derivation sample. The purpose of this study was to establish consensus among clinicians and experts on key predictors for persistent shoulder pain three months after initial consultation in primary care and assess the predictive performance of a model based on clinical expertise compared to a statistically derived model.

**Methods:**

A Delphi poll involving 3 rounds of data collection was used to reach consensus among health care professionals involved in the assessment and management of shoulder pain.

**Results:**

Predictors selected by the expert panel were: symptom duration, pain catastrophizing, symptom history, fear-avoidance beliefs, coexisting neck pain, severity of shoulder disability, multisite pain, age, shoulder pain intensity and illness perceptions. When tested in a sample of 587 primary care patients consulting with shoulder pain the predictive performance of the two prognostic models based on clinical expertise were lower compared to that of a statistically derived model (Area Under the Curve, AUC, expert-based dichotomous predictors 0.656, expert-based continuous predictors 0.679 vs. 0.702 statistical model).

**Conclusions:**

The three models were different in terms of composition, but all confirmed the prognostic importance of symptom duration, baseline level of shoulder disability and multisite pain. External validation in other populations of shoulder pain patients should confirm whether statistically derived models indeed perform better compared to models based on clinical expertise.

## Background

A clinical prediction rule is a simple tool which uses a combination of early signs or symptoms to provide a quantitative estimate of the absolute risk of particular outcomes for individual patients. Often the outcome is the individuals' expected course of an illness (prognosis), however clinical prediction rules can also be developed for predicting presence of a disease (diagnosis) or for predicting an individuals' response to a particular intervention[1]. The obtained estimations may subsequently be used by clinicians for the provision of patient information or to support decisions regarding treatment and referral.

Before a prediction rule can be implemented in clinical practice it ideally needs to be developed, validated and analysed for impact[1]. In prognostic research, prediction rules are generally derived by logistic or Cox regression models. With these statistical models, predictors are selected from a larger pool of potential predictors which is frequently established prior to model derivation and originates from previous literature or expert recommendations[1]. Selection is frequently based on forward or backward regression analysis in combination with a predefined p-value. However, it is not uncommon that prediction models derived by these methods perform poorly[2], with composition and predictive performance strongly dependent on characteristics of the derivation dataset. Especially for the prediction of non-specific musculoskeletal symptoms, the identification of good prediction models has appeared to be difficult[3]. In order not to miss potential predictors, prognostic researchers tend to gather an excessive amount of data, after which a smaller set of predictors is selected using statistical methods. Many prognostic models, however, especially in the area of musculoskeletal conditions consists of studies incorporating small sample sizes that are not in agreement with the suggested potential predictor to subject ratio[4] required for subsequent statistical analyses. Under these conditions, predictor selection by using statistical methods is known to yield unstable results independent of the strength of the association between predictor and outcome[2]. This may hamper the derivation of clinical useful prediction models with good practical performance and as a result has potential to be associated with invalid results in subsequent analysis (e.g., model validation).

When statistically deriving a prediction model, the contribution of clinical expertise prior to model derivation is often minimal. As a result, potential predictors obvious to clinicians might be overlooked. Therefore, the incorporation of clinical knowledge in the early phase of predictor selection can be of great importance. A technique known as a Delphi procedure[5], is believed to be an effective and reliable way of obtaining expert-based knowledge[6,7] and can be applied in prognostic research[8,9]. In this procedure, a group of experts responds anonymously to a series of subsequent questionnaires. Results are fed back to the panel in order to reach consensus. A potential advantage of this is that through the anonymous nature of the Delphi, negative group interactions (e.g., dominant group members forcing their beliefs onto the entire group) are eliminated[5].

In the present study we aimed to reach consensus among clinicians and experts regarding predictors that are most important for predicting persistent shoulder pain three months after initial consultation in primary care. A Delphi procedure with an expert panel of health care professionals involved in the assessment and management of shoulder pain was used to identify these key predictors. In a first step to determine the quality of these expert-based selected predictors two clinical/expert-based prognostic models were constructed and their predictive performance was compared with a third statistically derived prognostic model. Differences in model composition between the statistical and clinical models were expected and since the statistical model was modelled for the chosen dataset, predictive performance was expected to be better for the statistical model. These results will allow us to comment on whether the clinical models are an appropriate comparator model for future studies investigating predictive performance in a new sample (e.g., where statistical models often falter).

## Methods

### Delphi procedure

#### Expert panel selection

A multidisciplinary panel with members involved in or having thorough knowledge of shoulder pain in clinical practice was formed. We invited general practitioners, physiotherapists, orthopaedists and manual therapists from the United Kingdom as well as from the Netherlands to participate in the Delphi study. Expertise was defined by a) membership of a professional organisation combined with specific expertise in shoulder conditions (e.g., members of the U.K. Primary Care Rheumatology Society or the Dutch College of General Practitioners), b) being involved in guideline development or clinical research on shoulder pain or c) having a special interest and significant experience in treating shoulder conditions.

In order to obtain reliable results, a Delphi panel minimally needs to consist of 10 to 15 experts[10]. More participants will add to the reliability, but will complicate the procedure. We aimed to compose an expert panel of 20 members, a number which is commonly seen in consensus based research[11]. Accounting for non-response, we approached 52 experts in the area of shoulder symptoms. All were provided with an information letter explaining the aims, procedures and requirements of the Delphi study.

#### First round: Ranking potential predictors according to their predictive abilities

Similar to previous prognostic consensus studies[8,9], first a list of potential predictors based on a systematic review[12] was composed. In the first round, all panel members were presented with this list, which was sub-grouped in 7 categories (demographic, general health, characteristics of the shoulder symptom, pain related, psychological, social and physical load and activity). The panel members were asked to score the importance of each potential predictor on a 5 point Likert scale (i.e., 1 = not at all predictive, to 5 = highly predictive). When panel members felt that important predictors were missing from the provided list, they were encouraged to suggest additional potential predictors. Based on a summation of these scores all potential predictors were ranked according to their predictive ability. Newly suggested predictors were added to the list and arranged by the frequency with which they were suggested.

#### Second round: The re-evaluation of predictive abilities

The panel received feedback on the results of round one, and was subsequently asked in round two to rank the 10 most important potential predictors. This was done by rewarding the strongest predictor with 10 points and the weakest with 1 point. Hence all potential predictors were re-evaluated and arranged according to their predictive performance (i.e., the total of points rewarded to each potential predictor).

#### Third round: Consensus on the 10 most important predictors

In this third round panel members were asked whether or not they agreed on the 10 most important predictors from the second round. In case of disagreement, panel members were able to alter the selection by replacing a maximum of 3 predictors. Predictors could be eliminated from the selection or be replaced by others. In order to reduce the replacement options and come to consensus more easily, predictors could only be replaced by the 20 most predictive factors from round two. When eliminating or replacing any predictor, panel members were asked to provide a rationale for their decision. To reach consensus we a priori determined that at least 90% of the panel had to agree on all the predictors selected. If predictors were replaced or changed, participant consensus on the updated predictors was re-evaluated as part of round three. Based on argumented alterations a new selection was formed (i.e., predictors with <90% inclusion agreement were replaced by the most frequently mentioned replacement options) and together with these arguments were presented again for consensus.

### Predictive performance of the expert-based model

#### Data

The prediction models that were comprised of the predictors selected by our expert panel were evaluated and compared to a previously derived statistical prognostic model[13] using an existing dataset. Data used for this purpose came from the Dutch Shoulder Study[14], a cohort of 587 subjects consulting with a new episode of shoulder pain (i.e., had not consulted the GP or received treatment for the current shoulder problem in the previous three months) in general practice. Exclusion criteria of this cohort were: severe physical or psychological conditions (i.e., fractures or luxation in the shoulder region, rheumatic disease, neoplasms, neurological or vascular disorders, dementia) or cognitive limitations which would hinder the completion of a written questionnaire. The study was approved by the Medical Ethics Committee of the VU University Medical Centre, Amsterdam, The Netherlands.

#### Outcome

Persistent shoulder pain was defined by subtracting baseline scores (numeric scale 0-10) from follow-up scores. Subjects who improved less than 50% were indicated as having persistent shoulder pain. This definition of persistence was previously shown to be the minimal important change and was therefore used as cut-off value[13]. Outcome was measured at three months after initial consultation by a postal questionnaire.

#### Variables in the dataset

Collected data included demographic, shoulder pain related, physical and psychosocial factors, which were on average recorded 10 days after initial primary care consultation. Next to single questions, validated questionnaires were used to gather data[14]. Questionnaires used were; the Shoulder Disability Questionnaire (SDQ[15]) to assess the shoulder symptom severity (potential range: 0-100 points), the Pain Coping and Cognition List (PCCL[16]) to measure coping with pain (1-6 points), catastrophizing (1-6 points), internal (1-6 points) and external locus of control (1-6 points), the Four-Dimensional Symptom Questionnaire (4DSQ[17]) to assess anxiety (0-24 points), depression (0-12 points), distress (0-32 points) and somatisation (0-32 points), the physical activity scale of the Fear-Avoidance Beliefs Questionnaire (FABQ-PA[18]) to measure physical activity related fear-avoidance (0-42 points) and the Tampa Scale for Kinesiophobia (TSK[19,20]) items no. 1 and 9 to assess kinesiophobia (0-12 points).

#### Analysis

In order to retrieve information of all expert-based selected predictors, individual questionnaire items had to be used or combined. Consequently, with this information, we explored two different possibilities to derive a clinical/expert-based prognostic model. These methods will be compared and are explained below. Both clinical models consisted of the ten Delphi selected predictors, however predicted probabilities were obtained in different ways. Finally, the same data was used to create a statistically derived prognostic model to which both expert-based models were compared.

#### Expert-based model dichotomous: Clinical model including dichotomous expert selected predictors

In this model the ten predictors from our Delphi procedure were included as dichotomous predictors by using their median score as the split value. Subsequently regression coefficient estimates were derived by performing a multivariable logistic regression analysis.

#### Expert-based continuous: Clinical model including continuous expert selected predictors

For this model the same steps were conducted as for the derivation of expert model dichotomous. As dichotomizing can lead to information loss[21,22], expert-based selected predictors were included as continuous predictors where possible. Only when predictors failed the linearity assumption predictors were categorised into three groups.

#### Statistical model: Statistically derived multivariable regression based model

In the statistically derived model, predictors were, in contrast to the expert-based models, selected based on significance of the association with the outcome (persistent shoulder pain at 3 months). This selection was preceded by checking the linearity assumption for all potential predictors and if necessary categorisation (three groups) or dichotomization of potential predictors was performed. Furthermore, variable selection was also preceded by checking for (multi)collinearity (Pearson's r). Since correlated variables may disturb predictor selection[23], in case of correlated variables (r ≥ 0.5) the most easily obtainable variable in clinical practice was obtained for further analyses. Because of the great number of variables, a univariable pre-selection (α ≤ 0.157, a significance level which is comparable with Akaike's Information Criterion[24]) was performed to reduce the number of variables. Subsequently, a multivariable regression analysis in combination with a backward selection strategy (α = 0.157), was performed to obtain the final model[13].

Since the derivation of a prognostic model can seriously be affected by missing data[25], we used a multiple imputation procedure (MICE, Multivariate Imputation by Chained Equations[26]) to impute missing values. All models were derived and tested for performance using imputed data. Predictive performance was determined by how well predicted and observed probabilities agreed (calibration indicated by the slope of the calibration plot), how well the models distinguished subjects with and without persistent symptoms (discrimination indicated by the Area Under the receiver operating Curve (AUC) for dichotomous outcomes), explained variance (indicated by Nagelkerke's R^2^) and a bootstrap estimation of how much the model performance will deteriorate when applied to new subjects (overoptimism)[27].

#### Software

A web-based questionnaire was used in order to perform the Delphi procedure (Examine software[28]). Model derivation and assessment of model performance was performed by using R software version 2.6.0. Logistic regression and the bootstrap internal validation procedure were performed in R by using the R-Design package.

## Results

### Expert panel

**Figure 1:**
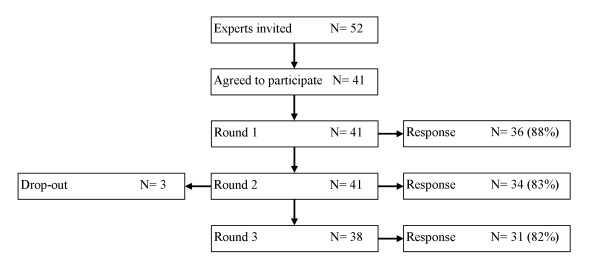
**Flowchart of panel member participation**.

52 Dutch and British health care professionals involved in the assessment and management of (patients with) shoulder disorders were invited to participate in our expert panel. From these 41 (79%) agreed to participate. The self reported primary professions indicated that among the participants were 16 (39%) general practitioners, 16 (39%) physiotherapists, 3 (8%) rheumatologists, 3 (8%) epidemiologists, 1 (3%) manual therapist and 1 (3%) senior lecturer in occupational medicine with a background as a GP and in occupational medicine. Half of all the participants combined their primary profession with a second vocation. From the participating physiotherapists 5 were also certified manual therapists and 3 GP's were professionally involved in musculoskeletal research. On average, the panel members had 17 (minimum of 5 and a maximum of 35) years of professional experience. Our international panel consisted of 25 (61%) British and 16 (39%) Dutch members. Figure 1 shows that participation of panel members varied from 88% to 82% in the separate Delphi rounds. 29 (71%) panel members completed all three Delphi rounds, 5 (12%) completed two rounds and 3 (7%) dropped out. All panel members contributing to the Delphi study are named in the acknowledgements.

### Delphi procedure

#### First round

In the first Delphi round we provided the expert panel with 46 potential predictors, which the experts ranked according to their predictive abilities. Table 1 presents the mean scores for predictive importance based on round 1. The highest scores were assigned to the variables symptom duration, symptom history and pain catastrophizing with mean (SD) score of respectively 4.3 (0.8), 4.1 (0.8) and 4.0 (0.9) as can be seen in Table 1. Mean scores (SD) for predictive importance ranged from 4.3 (0.8) for symptom duration to 2.0 (0.9) for coexisting knee pain or symptoms. Panel members suggested 19 additional potential predictors. These were added to the 46 listed potential predictors and separately arranged in the order of times they were suggested by individual panel members. Variables mentioned the most were (un)employment (mentioned by 12 panel members), high physical load at work or leisure time (mentioned by 11 panel members) and multisite pain (mentioned by 10 panel members).

**Table 1:** Delphi procedure results

	Round 1		Round 2		Round 3	
Predictors	mean (SD)	rank n = 46	sum score	rank n = 65	inclusion agreement	rank
Symptom duration	4.26 (0.78)	1	253	1	100%	1
Pain catastrophizing	4.03 (0.95)	3	160	2	84%	2
Symptom history	4.06 (0.80)	2	95	3	90%	3
Fear-avoidance beliefs	3.91 (1.04)	6	86	4	90%	4
Coexisting neck pain	3.49 (1.12)	22	79	5	100%	5
Baseline severity of shoulder disability	3.49 (1.07)	21	78	6	97%	6
Coexisting psychological complaints/general mental health problems	3.83 (0.95)	9	73	7	84%	-^b^
Currently on sick leave because of shoulder pain	3.69 (0.87)	11	70	8	81%	-^c^
Multisite pain	n = 10^a^	-	68	9	84%	7
Somatisation	4.00 (0.97)	4	67	10	84%	-^d^
Age	3.58 (0.73)	17	64	11	19%	8
Shoulder pain intensity	3.49 (1.24)	23	64	12	13%	9
Illness perceptions	3.91 (1.01)	5	62	13	13%	10
Depression/depressive symptoms	3.91 (1.07)	7	61	14	10%	-
Passive coping strategies	3.63 (1.06)	15	60	15	6%	-
Repetitive movements	3.86 (0.81)	8	46	16	3%	-
High physical load at work or leisure time	n = 11^a^	-	39	17	6%	-
Strain or overuse due to usual activities in work or leisure time	3.43 (1.06)	25	32	18	10%	-
Patient reports stiffness of the shoulder	3.06 (0.97)	30	32	19	6%	-
Diabetes Mellitus	n = 2^a^	-	32	20	10%	-

Predictors (only the 20 most highly ranked from round 2) are ordered by their prognostic importance as assessed in the second Delphi round

mean = mean score for rating individual potential predictor importance, [1 (not at all predictive) - 5 (highly predictive)]

SD = standard deviation

rank = the order of appearance of potential predictors in individual Delphi rounds based on scores for prognostic importance

sum score = the sum of all scores for predictive importance for each potential predictor

inclusion agreement = consensus on the inclusion of potential predictors in the selection of the ten most important predictors for persistent shoulder pain intensity

^a ^additional potential predictor added by n panel members

^b ^potential predictor omitted from final selection since it overlaps with 'fear-avoidance' and the item is 'too general'

^c ^potential predictor combined with predictor 'severity of shoulder disability'

^d ^potential predictor omitted from final selection since it overlaps with 'multisite pain' and 'fear avoidance'

#### Second round

The list of potential predictors and additional variables from the first round was re-evaluated in the second Delphi round. Table 1 presents the ranking and sum scores based on round 2 for the 20 predictors with the highest rankings. The results show that two potential predictors were considered to be very important by the panel; symptom duration and pain catastrophizing. Some predictors that were indicated as being moderately predictive in the first Delphi round (mean score approximately 3.5), were re-evaluated as being more important in the second round (i.e., baseline severity of shoulder disability and coexisting neck pain). On the contrary, the predictors illness perceptions, depression and repetitive movements were in the second round re-evaluated as being of lesser importance than indicated in the first round. From the newly suggested variables only multisite pain was regarded as being highly predictive. Other newly suggested variables were not included in the selection of key predictors. Based on the second round the 10 most important predictors were: symptom duration, pain catastrophizing, symptom history, fear-avoidance, coexisting neck pain, baseline severity of shoulder disability, coexisting psychological symptoms, sick leave because of shoulder pain, multisite pain, somatisation.

#### Third round

When presented with the ten key predictors as indicated by the second Delphi round, 13 (42%) panel members agreed on this selection. The majority of the experts (58%) however, disagreed with these predictors being the ten most important predictors of persistent shoulder pain. There was uncertainty regarding 5 predictors (pain catastrophizing, coexisting psychological symptoms, sick leave because of shoulder pain, multisite pain, and somatisation) which, as can be seen in Table 1, were selected among the top 10 predictors by ≤85% of the panel members. The main reason for this disagreement was that these predictors were believed to overlap with other included predictors. For instance, the predictor coexisting psychological symptoms was said to overlap with the predictors pain catastrophizing and fear-avoidance; somatisation with multisite pain; and sick leave with baseline shoulder disability. To resolve this problem of overlapping, panel members provided replacement options, such as age, shoulder pain intensity and illness perceptions mentioned by respectively 6, 4 and 4 panel members. These results lead to a new selection of most important predictors which is shown in Table 1. Consensus on this selection was achieved after the third Delphi round. This final set of predictors for persistent shoulder pain three months after initial consultation in primary care, which was agreed on by 29 (97%) panel members (i.e., higher than our predetermined consensus threshold of 90%) included: symptom duration, pain catastrophizing, symptom history, fear-avoidance, coexisting neck pain, severity of shoulder disability, multisite pain, age, shoulder pain intensity and illness perceptions. How these predictors formed the expert-based dichotomous model and the expert-based continuous model can be seen in Table 2.

**Table 2:** Regression coefficients and odds ratios for both the dichotomous and continuous expert-based prognostic model for persistent shoulder pain

	Expert-based model, dichotomous predictors			Expert-based model, continuous predictors		
Predictors	**category**^**a**^	β (SE)	OR (95% CI)	Category	β (SE)	OR (95% CI)
						
Symptom duration	>11 weeks	0.654 (0.183)	1.92 (1.34 - 2.75)	<6 weeks^b^		
presence of the current shoulder pain				6-11 weeks	0.600 (0.236)	1.82 (1.15 - 2.89)
problem for a period of				>11 weeks	0.898 (0.213)	2.45 (1.61 - 3.73)
Pain catastrophizing	NRS (0-10) >4	0.549 (0.184)	1.73 (1.21 - 2.48)	NRS (0-10)	0.092 (0.032)	1.10 (1.03 - 1.17)
believing shoulder pain to be permanent rather than temporary						
Symptom history	yes	0.188 (0.181)	1.21 (0.85 - 1.72)	yes	0.210 (0.185)	1.23 (0.86 - 1.77)
experienced earlier episode(s) of shoulder pain						
Fear-avoidance beliefs	NRS (0-10) >7	-0.031 (0.180)	0.97 (0.68 - 1.38)	NRS (0-10)	-0.003 (0.028)	0.99 (0.94 - 1.05)
believing activity will worsen the shoulder pain						
Coexisting neck pain	yes	-0.067 (0.207)	0.93 (0.62 - 1.40)	yes	-0.066 (0.211)	0.94 (0.62 - 1.41)
additional neck pain during the current shoulder pain period						
Severity of shoulder disability	yes	0.130 (0.178)	1.14 (0.80 - 1.61)	score (0-20)	-0.032 (0.021)	0.97 (0.93 - 1.01)
being unable to perform normal daily activities in the past week or for a longer period of time						
Multisite pain	yes	0.294 (0.219)	1.34 (0.87 - 2.06)	yes	0.378 (0.223)	1.46 (0.94 - 2.26)
pain or stiffness in other areas than the afflicted shoulder						
Age	>52 years	0.275 (0.176)	1.32 (0.93 - 1.86)	years	0.015 (0.007)	1.01 (1.00 - 1.03)
Shoulder pain intensity	NRS (0-10) > 5	-0.388 (0.186)	0.68 (0.47 - 0.98)	NRS (0-10)	-0.150 (0.044)	0.86 (0.79 - 0.94)
shoulder pain experienced in the last 24 hours						
Illness perceptions	yes	0.144 (0.177)	1.15 (0.81 - 1.63)	yes	0.148 (0.181)	1.16 (0.81 - 1.65)
believing there is not a lot the person can do to control the shoulder pain intercept		-1.078 (0.226)			-1.119 (0.565)	

Predictors from the third Delphi round together with their suggested formulations as indicated by the expert panel

B = regression coefficient estimate

SE = standard error of regression coefficient estimate

OR = odds ratio

95% CI = 95% confidence interval for the odds ratio

^a ^predictors were dichotomized by using median split value scores

^b ^reference category

### Statistically derived model

As can be seen in Table 3 the statistically derived model included the following predictors: sporting injury, symptom duration, coexisting lower back pain, bilateral shoulder pain, inability to perform daily activities and coexisting upper extremity pain. Only two of these were included in the expert-based selected predictors; symptom duration and baseline severity of the shoulder disability (included in the statistical model as the inability to perform daily activities). However, other statistically selected predictors seem to reflect the expert selected predictor multisite pain (i.e., coexisting back pain, bilateral shoulder pain and upper extremity pain).

**Table 3:** Regression coefficients and odds ratios for the statistical prognostic model for persistent shoulder pain

	Statistical model		
Predictors	category	β (SE)	OR (95% CI)
Sporting injury	yes	-1.228 (0.499)	0.29 (0.11 - 0.79)
Longer symptom duration	<6 weeks^a^		
	6-11 weeks	0.514 (0.253)	1.67 (1.01 - 2.75)
	>11 weeks	0.922 (0.230)	2.51 (1.60 - 3.96)
Coexisting lower back pain	yes	0.915 (0.233)	2.50 (1.57 - 3.96)
Bilateral shoulder pain	yes	0.706 (0.298)	2.03 (1.12 - 3.65)
Inability to perform daily activities	0 days^a^		
	1-30 days	-0.552 (0.220)	0.57 (0.37 - 0.98)
	1-12 months	-0.431 (0.342)	0.65 (0.33 - 1.29)
Coexisting upper extremity pain	yes	0.340 (0.204)	1.40 (0.94 - 2.10)
intercept		-0.770 (0.201)	

B = regression coefficient estimate

SE = standard error of regression coefficient estimate

OR = odds ratio

95% CI = 95% confidence interval for the odds ratio

^a ^reference category

### How well do our models predict persistent shoulder pain at 3 months

**Figure 2:**
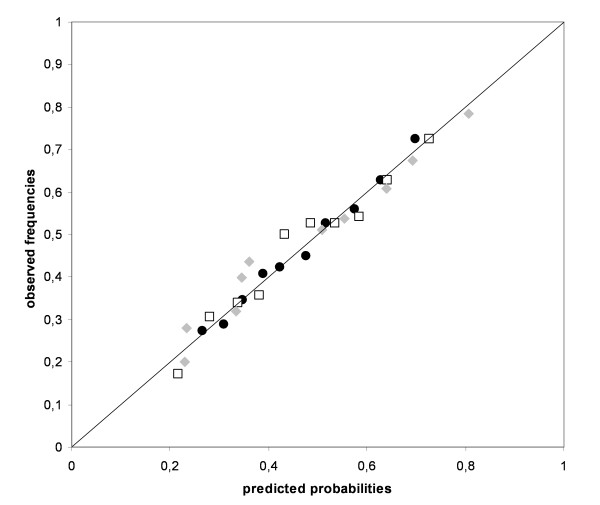
**Calibration plot for both the expert-based and statistically derived models for the prognosis of persistent shoulder pain**. Closed black dots = Expert model with dichotomized predictors Open squares = Expert model with continuous predictors Closed grey diamonds = Statistical model

Figure 2 shows the agreement between observed and predicted probabilities for both the statistical and expert-based models in a calibration plot. Following application in the dataset, Figure 2 showed that the predicted probabilities for the expert model with dichotomous predictors ranged from 0.18 to 0.76 and from 0.15 to 0.84 for the expert model with continuous predictors. The statistically derived model showed a similar range with predicted probabilities between 0.12 and 0.89. Since each model was fitted on the derivation data by multivariable regression analysis, all showed good calibration with calibration points close to the diagonal (i.e., optimal calibration). No differences in calibration slopes between statistical (1.019) and expert-based (1.017 dichotomous and 0.986 continuous) models were observed (Table 4). Table 4 also shows that the expert-based models had lower discriminative abilities compared to the statistical model (AUC expert dichotomous model = 0.656, AUC expert continuous model = 0.679 and AUC statistical model = 0.702). Hence the statistical model distinguishes better between subjects with and without persistent shoulder pain than the expert-based models. Although the calibration plot showed all models to be well fitted on the derivation data, the internal validation routine showed that regression coefficient estimates of all three models were overoptimistic. This means that when applied in new subjects, the predictive performance of all three models is expected to deteriorate[25]. With bootstrap estimated rates for overoptimism of 0.029 and 0.028, the regression coefficient estimates of the expert based models appeared to be twice as optimistic as the regression coefficient estimates of the statistical model (with an estimated overoptimism of 0.014). Therefore the statistical model is expected to perform better when applied in new subjects[27].

**Table 4:** Performance measures for the expert-based and statistically derived prognostic models

	Expert model dicho	Expert model cont	Statistical model
calibration slope	1.017	0.986	1.019
R^2^_N_	0.098	0.13	0.162
AUC	0.656	0.679	0.702
95% CI	0.612 - 0.700	0.636 - 0.722	0.660 - 0.745
Optimism	0.029	0.028	0.014
AUCcorrected	0.627	0.651	0.688

Expert model dicho = prognostic model using dichotomized expert selected predictors

Expert model cont = prognostic model using continuous expert selected predictors

R^2^_N _= Nagelkerke's explained variance

AUC = Discriminative ability as indicated by the Area Under the ROC Curve

95% CI = 95% confidence interval for the AUC

Optimism = the models' estimated deterioration when applied to new subjects

AUCcorrected = the optimism corrected Area Under the ROC Curve

## Discussion

This Delphi procedure resulted in professional consensus on the 10 most important predictors of persistent shoulder pain 3 months after initial consultation in primary care. Expert selected predictors appeared to be different from that of a statistically derived model, however both models confirmed the importance of symptom duration, baseline level of disability and multisite pain. Panel members additionally selected age, baseline pain intensity and psychological factors as important predictors. Concerning predictive performance, we found the statistically derived model to be slightly better than the expert-based prognostic model.

Since clinical expertise is expected to complement statistically derived prognostic models, this study aimed to reach clinical consensus on which predictors are most important for predicting persistent shoulder pain. It was shown that health care professionals' consensus based selection of key predictors reflected most statistically selected predictors but also included additional predictors which were not identified by statistical selection. During the inventory of potential predictors (i.e., the first Delphi round) health care professionals even identified predictors which previously have not been directly associated with shoulder pain together with predictors which have been shown to be associated with poor outcome in other musculoskeletal pain conditions[29] (e.g., earlier experiences with shoulder treatment, smoking, diabetes mellitus, alcohol intake, ethnicity, level of training discipline, perceived versus actual work activity, social support, distress). None of these predictors made it to the final selection of most important predictors.

The consensus based selection of the key predictors of persistent shoulder pain, was derived using a Delphi procedure. Although this technique is often applied in consensus based research[5,30], its validity and reliability are sometimes object of discussion[31]. Since consensus findings may vary depending on the panel, the guidelines for consensus methods by Fink et al.[32] were followed where possible. With a minimum participation rate of 31 panel members in a single Delphi round, our expert panel was sufficiently sized for obtaining reliable results[10]. As multi-disciplinary panels may select a wider range of predictors compared to single-disciplinary panels[33,34], our panel consisted of health care professionals and researchers from different disciplines and geographical areas in the United Kingdom and the Netherlands. Furthermore, the Delphi procedure was completely anonymous. Panel members never met, neither did they knew each others identities. Therefore, negative group interactions or dominant opinions were eliminated. To assist our panel members in selecting prognostic factors we provided them with a resource, i.e., a list of potential predictors based on a previous systematic review[12]. Although not an uncommon practice in consensus based research[8,9], one might argue that providing such a list might hinder the unveiling of new potential predictors. Therefore, during the entire Delphi process all panel members were encouraged to suggest additional potential predictors. Since a part of our panel was also involved in shoulder related clinical research, they were considered to be informed on the latest developments in the literature. This together with the option of providing additional information lead us to believe all predictors for persistent shoulder pain in primary care patients were identified by our panel.

How can we explain observed differences in expert and statistical selected prognostic factors? Taking into account the above mentioned considerations, it is unlikely that these differences were caused by methodological limitations in the Delphi procedure. Because our panel of health care professionals was trained in the clinical management of individual patients, they might have had problems with providing prognostic factors for the general population of shoulder pain patients. This could have complicated the identification of universal prognostic factors for shoulder pain patients. Another explanation for the observed differences in selected predictors might be found in the methodological limitations of predictor selection in statistically derived models. In the applied methodology, predictors were selected by an automated selection procedure. As shown by Austin and Tu[2], statistical predictor selection can give biased results. Automated backward elimination or forward selection might result in omission of important predictors or the random selection of less important predictors. As a result statistically derived models may be unstable[2], which was previously demonstrated for our statistically derived model[13]. Differences between expert-based and statistical selection of predictors might therefore be largely influenced by the chosen method of statistical predictor selection. However, how to optimally perform variable selection is still a subject of discussion[35].

One of the strengths of the current study was that next to establishing consensus on key predictors, the predictive performance of these predictors was empirically tested. Results showed that both expert-based models did not perform as optimally compared to the statistically derived prognostic model. This is a notable result since clinical knowledge is expected to complement statistical modelling and the derivation of our statistical model has some known limitations in predictor selection. These findings do however need to be interpreted with caution since they do not suggest that statistical based scoring systems are superior to clinical prognosis. Although we asked our panel for suggestions on how to formulate and score each predictor, a weakness of this study was that we had to use an existing dataset which did not include the exact same variables as proposed by the expert panel. Another weakness was that a potential floor-effect associated with low baseline pain ratings could have occurred in our measure of outcome. Although approximately 19% of the subjects in our database had a baseline pain score of ≤2, all baseline pain categories (e.g., 1 to 10) showed a constant percentage of subjects identified with persistent shoulder pain of approximately 40 to 60%. Thus, apart from subjects with a baseline pain rating of 0 we reasoned that our analyses were not affected by a potential floor effect. Furthermore, although we derived an optimal model using continuous scales, the expert-based model had to compete with a statistical model that was derived in the same dataset and therefore was expected to show better predictive performance. Hence the conclusion of the superiority of statistical prognosis over clinical prognosis might be impetuous. Another aspect that can be regarded as a weakness of our study is the dichotomization of key predictors in one of the expert-based prognostic models. Dichotomization of predictors is in the literature often criticized because it may lead to loss of information and thus a decrease in predictive performance[22]. Although we expected our panel members to be familiar with this undesirable effect, most of them said they preferred a prognostic model which consists of simple (i.e., dichotomous) predictors. This illustrates at this point the discrepancy between prognostic research and clinical practice. In prognostic research model performance is most important, in clinical practice models in addition need to be easy to use. Unfortunately simplicity of the model goes at cost of the predictive performance, as can be seen by the effect of dichotomisation of predictors by using median values as cut-off[21,22].

With these considerations it remains unclear whether estimations of prognosis by health care professionals are superior or not to the estimation of prognosis obtained by scoring systems. Previous studies have shown that both clinical prognosis and scoring systems can be superior to one another[36-38]. It might even be conceivable that prognostic superiority is case dependent (type of musculoskeletal condition, health care profession). Therefore, clinical prognosis and scoring systems for the prognosis of non-recovery from shoulder pain will be compared in a future study.

## Conclusions

As clinical expertise is expected to complement statistically based prognostic research we showed that an expert panel of health care professionals and researchers indeed selected additional predictors compared to a statistical selection procedure, emphasizing the expected value of age, shoulder pain intensity and psychological factors. Both selections confirmed the importance of symptom duration, baseline severity of shoulder disability and multisite pain in the prognosis of shoulder pain. When transformed to a prognostic model, the expert-based models performed less well compared to a statistically derived model. Application in a different population than the derivation dataset should demonstrate whether statistically derived models are indeed less overoptimistic compared to expert-based models.

## Competing interests

The authors declare that they have no competing interests.

## Authors' contributions

DAWMvdW was responsible for the conception and design of the study and helped to design the study's analytic strategy. MWH and HCWdV helped to prepare the Methods and the Discussion sections. HEvdH, HCWdV, MWH and DAWMvdW critically appraised and revised draft versions of the manuscript. DV designed and conducted the Delphi procedure, prepared the data for analysis, designed the analytic strategy, conducted all analyses and wrote the paper. All authors read and approved the final manuscript.

## Pre-publication history

The pre-publication history for this paper can be accessed here:

http://www.biomedcentral.com/1471-2296/12/63/prepub
